# Prognostic significance of neutrophil-lymphocyte ratio in hepatocellular carcinoma: a meta-analysis

**DOI:** 10.1186/1471-2407-14-117

**Published:** 2014-02-21

**Authors:** Wei-Kai Xiao, Dong Chen, Shao-Qiang Li, Shun-Jun Fu, Bao-Gang Peng, Li-Jian Liang

**Affiliations:** 1 Department of Hepatobiliary Surgery, the First Affiliated Hospital, Sun Yat-sen University, No. 58 Zhongshan Er Road, Guangzhou 510080, China

**Keywords:** Neutrophil-lymphocyte ratio, Hepatocellular carcinoma, Prognosis

## Abstract

**Backgrounds:**

Neutrophil-lymphocyte ratio (NLR) has recently been reported as a predictor of Hepatocellular carcinoma (HCC). However, its prognostic value in HCC still remains controversial. In this study, we aimed to evaluate the association between NLR and clinical outcome of HCC patients by performing meta-analysis.

**Methods:**

A comprehensive literature search for relevant studies published up to August 2013 was performed by using PubMed, Ovid, the Cochrane Library and Web of Science databases. Meta-analysis was performed using hazard ratio (HR) or odds ratio (OR) and 95% confidence intervals (95% CIs) as effect measures.

**Results:**

A total of 15 studies encompassing 3094 patients were included in this meta-analysis. Our pooled results showed that high NLR was associated with poor overall survival (OS) and disease free survival (DFS) in HCC initially treated by liver transplantation (HR = 3.42, 95% CI:2.41-4.85,P = 0.000; HR = 5.90, 95% CI:3.99-8.70,P = 0.000, respectively) and surgical resection (HR = 3.33, 95% CI:2.23-4.98, P = 0.000; HR = 2.10, 95% CI: 2.06–2.14, respectively). High NLR was also associated with poor OS in HCC treated by radiofrequency-ablation (HR = 1.28, 95%CI: 1.10-1.48, P = 0.000), TACE (HR = 2.52, 95% CI: 1.64-3.86, P = 0.000) and mixed treatment (HR = 1.85, 95%  CI: 1.40-2.44, P = 0.000), respectively. In addition, high NLR was significantly correlated with the presence of vascular invasion (OR = 2.69, 95% CI: 2.01–3.59, P = 0.000), tumor multifocality (OR = 1.74, 95% CI: 1.30–2.34, P = 0.000) and higher incidence of AFP ≥ 400 ng/ml (OR = 1.46, 95% CI: 1.01–2.09, P = 0.04).

**Conclusion:**

Elevated NLR indicates a poor prognosis for patients with HCC. NLR may be a convenient, easily-obtained, low cost and reliable biomarker with prognostic potential for HCC.

## Background

Hepatocellular carcinoma (HCC) is the sixth most common malignant tumors worldwide with increasing incidence rate over the last several decades across the world. Meanwhile, its third cancer-related mortality among varieties of cancers indicates the poor prognosis of HCC [1]. Despite a significant improvement of HCC management, including liver transplantation(LT), surgical resection, radiofrequency ablation(RFA), transarterial chemoembolization(TACE) and molecular therapy has been achieved, the long-term outcome is still disappointing owing to high recurrence and high fatality of the disease [2]. Thus, there is an urgent need for us to identify better prognostic biomarkers, especially serum biomarkers for prognosis and metastatic recurrence of HCC, which would help clinicians to adopt preventive and therapeutic strategies for risk patients.

In recent years, accumulating evidence demonstrated that increased systemic inflammation is associated with poor cancer-specific survival in a variety of cancers [3-7]. These studies revealed that the host’s inflammatory response to cancer and/or the systemic effects exerted by the cancer cells leads to upregulation of the inflammatory process, inducing the proliferation and metastasis of cancer cells by inhibiting apoptosis, promoting angiogenesis, and repairing DNA damage [8,9]. The presence of a systemic inflammatory response can be detected by both the elevation of the C-reactive protein (CRP) level [10] and neutrophil-lymphocyte ratio (NLR) [11].

A high preoperative serum CRP level has been found to be associated with early recurrence of HCC and poorer survival after hepatic resection [12], but CRP is not routinely measured in many hospitals and CRP level displays nonspecific change after treatment [13]. Several studies have shown that an elevation in NLR correlated with tumor progression, metastasis, and clinical outcome in a variety of tumors besides HCC [14-18]. Nevertheless, conflicting data have emerged regarding the ability of NLR to predict disease progression and overall survival (OS) in HCC. Therefore, it is necessary to perform a meta-analysis to systematically and comprehensively understand the prognostic value of NLR in HCC.

In this study, we aimed to assess the prognostic significance of high NLR for overall survival (OS) and disease-free survival (DFS) for HCC by pooling outcomes from the available data. In addition, the correlation between NLR and patients’ clinicopathological features was also examined.

## Methods

### Identification and selection of studies

#### Study objectives

The primary endpoint was to evaluate patients’ OS and DFS based on their NLR profiles. The secondary endpoint was to assess the relation between NLR and patients’ clinicopathological features (such as vascular invasion).

#### Search strategy

The following databases were systematically searched in August 2013 without time restrictions: PubMed, Ovid, the Cochrane Library and Web of Science databases. The search strategy was based on combinations of the following terms: (NLR or neutrophil-lymphocyte ratio) AND (HCC or hepatocellular carcinoma). Reports in English were eligible for inclusion. The reference list was also checked for relevant articles. Investigators were contacted and asked to supply additional data when key information relevant to the meta-analysis was missing.

#### Inclusion criteria of studies

All studies included in this meta-analysis must meet the following criteria: (1) NLR was measured by serum-based methods; (2) The relationship between NLR and OS and/or DFS of patients with HCC was evaluated; (3) Sample size was greater than 20.

#### Definitions and data extraction

NLR was defined as the serum absolute neutrophil count divided by the serum absolute lymphocyte count in peripheral blood [19]. OS was defined as the interval between the medical treatment including liver resection, liver transplantation or radiofrequency ablation (RFA), etc. and the death or the last observation of patients. DFS was measured from the date of curative treatment until the detection of tumor recurrence. Tumor vascular invasion was defined as presence of either macro- or microscopic vascular invasion (including portal vein invasion, hepatic vein invasion.). Tumor multifocality was defined as tumor number greater than 2 or 3. The histologic grade of tumor was assigned according to the Edmondson Steiner grading system, studies were grouped as well/moderate (I/II) or poor (III/IV) degrees of differentiation. All data extractions were performed separately by X.W.K. and C.D. Disagreements were resolved by discussion.

#### Qualitative assessment

The quality assessment of included studies was evaluated by the modified Newcastle–Ottawa quality assessment scale for cohort studies [20,21] (see “Newcastle-Ottawa quality assessment scale” section). This scale consists of three factors: patient selection, comparability of the study groups, and assessment of outcomes. A score of 0-9 (labeled as stars) was used to indicate the quality of each study. Studies labeled with six or more stars were considered to be of high quality.

##### Newcastle-Ottawa quality assessment scale

Selection

(1) Representativeness of the exposed cohort

(a) Truly representative of the average HCC patients in the community*

(b) Somewhat representative of the average HCC patients in the community*

(c) Selected group of users (e.g., nurses, volunteers)

(d) No description of the derivation of the cohort

(2) Selection of the non exposed cohort

(a) Drawn from the same community as the exposed cohort*

(b) Drawn from a different source

(c) No description of the derivation of the non exposed cohort

(3) Ascertainment of exposure (Proof of HCC and NLR measurement)

(a) Secure record (e.g., surgical records)*

(b) Structured interview*

(c) Written self report

(d) No description

(4) Demonstration that outcome of interest was not present at start of study

(a) Yes*

(b) No

Comparability

(1) Comparability of cohorts on the basis of the design or analysis

(a) Study controls for recurrence or metastasis*

(b) Study controls for any additional factor (Age, gender, grade, alpha-fetoprotein level, etc.)*

Outcome

(1) Assessment of outcome

(a) Independent blind assessment*

(b) Record linkage*

(c) Self report

(d) No description

(2) Was follow-up long enough for outcomes to occur? (Death or recurrence)

(a) Yes (3 years)*

(b) No

(3) Adequacy of follow up of cohorts

(a) Complete follow up – all subjects accounted for*

(b) Subjects lost to follow up unlikely to introduce bias – small number lost – (25%) follow up, or description provided of those lost)*

(c) Follow up rate (<75%) and no description of those lost

(d) No statement

A study can be awarded a maximum of one star (*) for each numbered item within the Selection and Outcome categories. A maximum of two stars can be given for Comparability. Underlined and quoted phrases are provided in the scale to allow for adjustment to particular studies. Italicised phrases indicate our interpretation of the question relevant to this study.

### Quantitative analysis (meta-analysis)

#### Statistical methods

Included studies were divided into two groups for analysis: those with OS data and those with DFS. Data on the prognostic ability of NLR for OS and DFS were pooled across studies. For the quantitative aggregation of the survival results, hazard ratios (HRs) and their associated standard errors (SEs) were pooled to give the effective value. When these statistical variables were not directly provided in the original articles, they were calculated from available numerical data using methods reported by Parmar et al. [22]. For the pooled analysis of the relation between NLR and clinicopathological features (such as vascular invasion), odds ratios (ORs) and their 95% confidence intervals (95% CIs) were pooled to give the effective value.

In this study, the cut-off value for high or low NLR was determined by investigators of each study. A uniform NLR was not obtained in this study. A HR >1 implies a worse prognosis in the group with high NLR; while an OR > 1 indicated higher probability for high tumor grade, later tumor stage or the presence of vascular invasion in the group with High NLR. The point estimate of the HR or OR was considered statistically significant at the *p* < 0.05 level if the 95% CI for the overall HR did not overlap one. In the course of data pooling, we used the I-squared (I^2^) statistic to measure the extent of inconsistency among the results and tested the heterogeneity using chi-square (χ^2^) test. Because this test has poor power in the case of few studies, we considered both the presence of significant heterogeneity at the 10% level of significance and values of I^2^ exceeding 56% as an indicator of significant heterogeneity [23]. The random-effects model was used if there was heterogeneity between studies; otherwise, the fixed-effects model was used. Analysis on main results was performed by using Review Manager Version 5.0 software (Copenhagen: The Nordic Cochrane Centre; The Cochrane Collaboration, 2008).

## Results

### Selection and characteristics of studies

88 records were identified regarding the association of NLR and HCC via the initial literature search. 72 studies were excluded after screening the titles or abstracts as they were either review articles, abstracts, experiment research, duplicate reports, reports in language other than English or studies irrelevant to the current analysis. After careful evaluation by applying our inclusion criteria, a total of 16 eligible studies were identified [24-39]. Of the 16 studies, two were reported by the same study center [28,30], and the patients were overlapping or partly overlapping in the studies. To avoid duplicate counting, only one study with more complete data was selected [30]. Therefore, 15 studies [24-27,29-39] with 3094 patients which met our inclusion criteria were selected for our meta-analysis finally. Three studies were performed in USA [27,29,38], Japan [24,30,31], China [25,34,37], and UK [32,33,39]_,_ respectively, one in Taiwan [35], Korea [26] and in Italy [36], respectively. Liver transplantation as initial treatment for HCC was reported in 5 studies [27,30,34,36,37]. Mixed treatment (including locoregional, systemic treatments or supportive care) [26,31,32] and TACE [29,33,37] were reported in 3 studies, respectively. Surgical resection [24,39] and radiofrequency ablation [25,35] were reported in 2 studies, respectively. Sample sizes ranged from 54 to 958. Mean or median age ranged from 48.4 to 72 years. The number of male population varied from 40 to 689. The number of HCC patients with vascular invasion ranged from 25 to 124. OS was reported or estimated in all studies, whereas DFS was only provided in nine studies [24,25,27,30,34-36,38,39].The scores of study quality assessed by Newcastle-Ottawa quality assessment scale ranged from 5 to 8 (with a mean of 6.73). A high value indicated better methodology. HRs were recorded for each study using available data or the methods described above. Individual study reported a “high” NLR with survival data, the NLR cut-off value was determined using different methods in each study. The basic features of the fifteen studies were summarized in Table 1.

**Table 1:** Baseline characteristics of the studies in the meta-analysis

**Study (Reference)**	**Year**	**Country**	**Treatment**	**Sample size(Male, n)**	**Mean/median Ages(years)**	**Tumor vascular invasion (yes)**	**NLR (cut-off used;n)**
**Halazun**[38]	2009	USA	LT	150(119)	57.1	45	5
**Motomura**[30]	2013	Japan	LT	158(92)	57	59	4
**Bertuzzo**[36]	2011	Italy	LT	219(186)	57	124	5
**Limaye**[27]	2013	USA	LT	160(130)	55.5/55.1	25	5
**Wang**[34]	2011	China	LT	101(92)	48.4	30	3
**Gomez**[39]	2008	UK	SR	96(72)	65	49	5
**Mano**[24]	2013	Japan	SR	958(689)	67	NA	2.81
**McNally**[29]	2013	USA	TACE	103(77)	56	NA	5
**Huang**[37]	2011	China	TACE	145(134)	49	39	3.3
**Pinato1**[33]	2012	UK	TACE	54(40)	63	NA	5
**Chen**[35]	2011	Taiwan	RF	192(95)	65.7	NA	2.4
**Dan**[25]	2013	China	RF	178(159)	57	NA	1.9
**Oh**[26]	2013	Korea	Mix	318((240)	58	107	2.3
**Pinato2**[32]	2012	UK	Mix	112(90)	65	NA	5
**Kinoshita**[31]	2012	Japan	Mix	150(106)	72	NA	5
**Study**	**Sampling time**		**Follow-up Mean/median (months)**	**Outcome measured**	**Multivariate analysis**	**Patients with elevated NLR**	**Study quality (Points)**
**Halazun**[38]	Pre-LT		37.2	DFS/ OS	yes	13	8/9
**Motomura**[30]	Pre-LT		40.3	DFS/OS	yes	26	8/9
**Bertuzzo**[36]	Pre-LT		40	DFS/ OS	yes	23	5/9
**Limaye**[27]	Pre-LT		38	DFS/ OS	yes	28	8/9
**Wang**[34]	Pre-LT		34.2	DFS/ OS	yes	33	7/9
**Gomez**[39]	Pre-SR		30	DFS/ OS	yes	26	6/9
**Mano**[24]	Pre-SR		NA	DFS/ OS	yes	238	5/9
**McNally**[29]	Pre-TACE		11.1	OS	no	18	7/9
**Huang**[37]	Pre- TACE		10	OS	yes	59	8/9
**Pinato1**[33]	Pre-TACE		NA	OS	Yes	9	5/9
**Chen**[35]	Pre-RFA		34	OS/DFS	yes	81	7/9
**Dan**[25]	Pre-RFA		52.7	OS/DFS	yes	91	7/9
**Oh**[26]	Pre-treatment		13.9	OS	yes	189	7/9
**Pinato2**[32]	Pre-treatment		10	OS	yes	25	7/9
**Kinoshita**[31]	Pre-treatment		18	0S	no	15	6/9

M, male; F, female; NA, not available.

Treatment describes whether the patients received surgical resection (SR), or liver transplantation (LT) of HCC, transarterial chemoembolization (TACE), radiofrequency ablation(RFA), mixed treatment (Mix) including locoregional ,systemic treatments or supportive care.

Tumor vascular invasion was defined as presence of either macro- or microscopic vascular invasion (including portal vein invasion, etc.).

OS, overall survival; DFS, disease-free survival.

Study quality is listed using the results of the Newcastle –Ottawa questionnaire.

### NLR and OS in HCC

**Figure 1:**
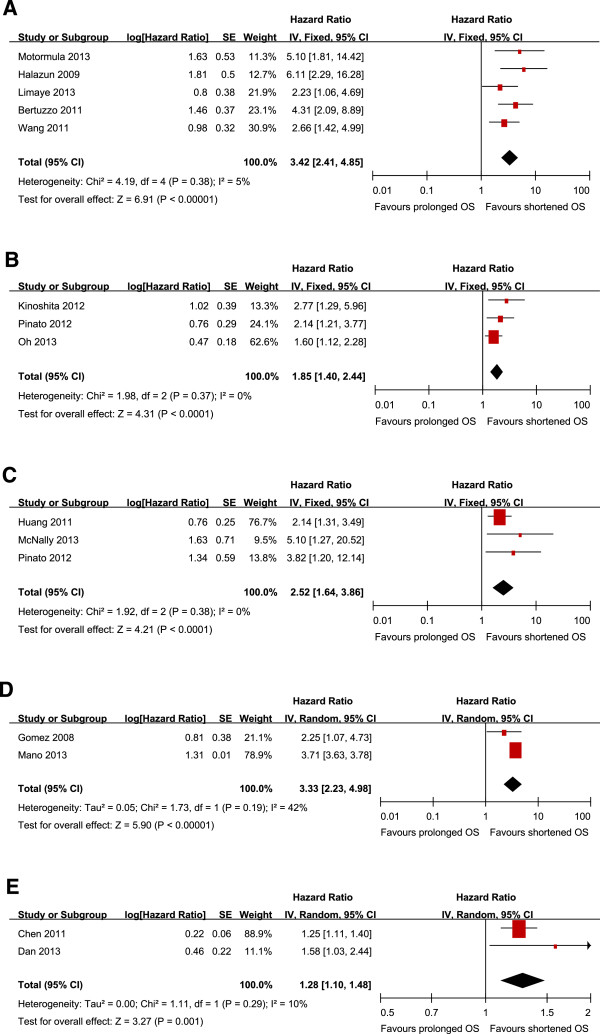
**Meta-analysis of the association between NLR and OS of HCC initially treated by liver transplantation (1A), mixed treatment (1B), TACE(1C), surgical resection(1D) and RFA(1E).** Results are presented as individual and pooled hazard ratio (HR), and 95% confidence interval (CI).

All the fifteen studies reported the relationship between NLR and OS in HCC. Five studies [27,30,34,36,38] presented the information of NLR correlated with OS in HCC initially treated by liver transplantation. Pooled data from these five studies showed that increased NLR were significantly correlated with poor OS with a pooled estimate HR of 3.42 (95% CI: 2.41–4.85, P = 0.000; Figure 1A), and without significant heterogeneity in the data (χ^2^ =4.19, I^2^ = 5%, P = 0.38). 3 studies reported data on mixed treatment and 3 on TACE, respectively. The pooled data showed that high NLR were significantly associated with poor OS of HCC initially treated by mixed treatment (HR = 1.85; 95% CI: 40-2.44, P = 0.000, Figure 1B) and TACE (HR: 2.52, 95% CI: 1.64–3.86, p =0.000; Figure 1C). There was no any heterogeneity in both treatment groups (χ^2^ =1.98, I^2^ = 0%, p =0.37), (χ^2^ =1.92, I^2^ = 0%, p =0.38). Finally, pooled outcomes show that NLR significantly correlated with OS in HCC initially treated by surgical resection (HR: 3.33, 95% CI: 2.23–4.98, P =0.000; Figure 1D), and RFA (HR: 1.28, 95% CI: 1.10–1.48, P = 0.001; Figure 1E).

### NLR and DFS in HCC

**Figure 2:**
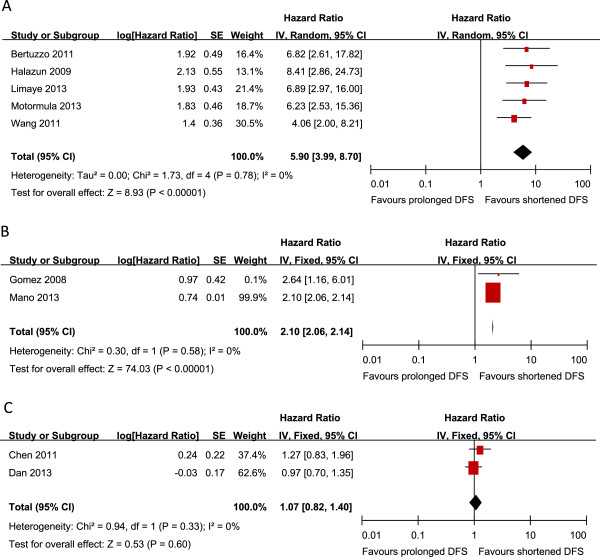
**Meta-analysis of the association between NLR and DFS of HCC initially treated by liver transplantation (2A), surgical resection(2B) and radiofrequency ablation (2C).** Results are presented as individual and pooled hazard ratio (HR), and 95% confidence interval (CI).

Nine studies reported data on NLR and DFS in HCC. Five studies offered data on NLR and DFS in HCC initially treated by LT [27,30,34,36,38]. Pooled data showed a significant correlation of increased NLR with poor DFS with a pooled HR estimate of 5.90 (95% CI: 3.99–8.70, P = 0.000; Figure 2A), and without any heterogeneity in the data (χ^2^ =1.73, I^2^ =0.0%, p = 0.78). Furthermore, pooled outcomes showed that NLR significantly correlated with DFS in HCC initially treated by surgical resection (HR: 2.10, 95% CI: 2.06–2.14, p =0.000; Figure 2B) without any heterogeneity (X^2^ =0.30, I^2^ = 0%, p =0.58). Finally, 2 studies provided data on radiofrequency ablation. But no correlation was observed between NLR and DFS (HR: 1.07, 95% CI: 0.82–1.40, p =0.60; Figure 2C).

### NLR and tumor pathologic features

**Figure 3:**
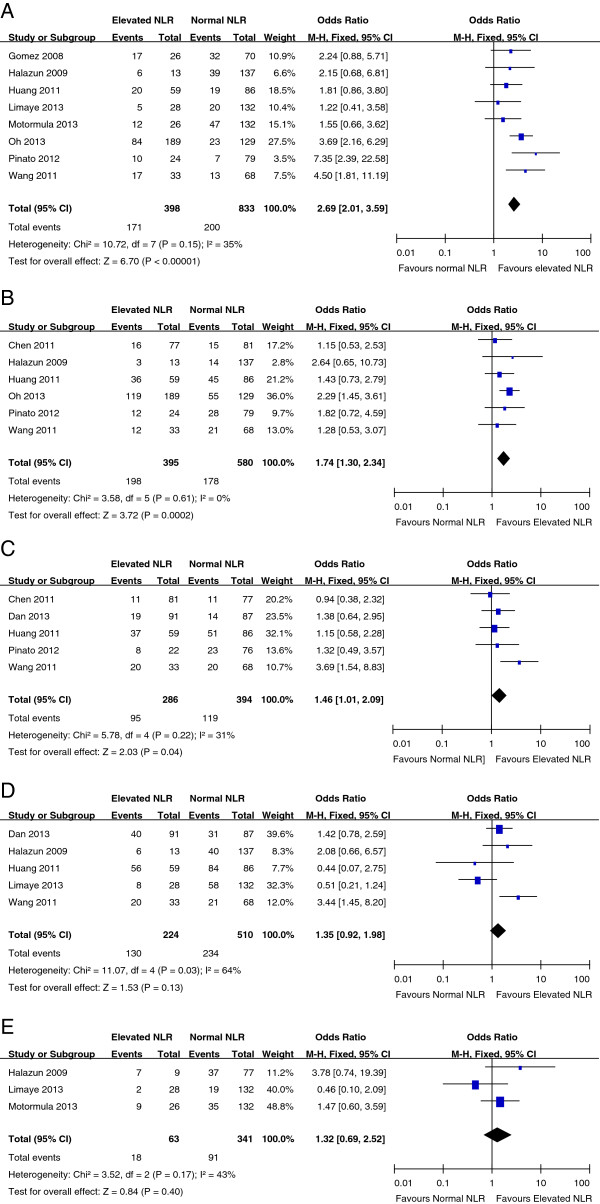
**Meta-analysis of the association between NLR and vascular invasion (3A), tumor multifocality (3B), incidence of AFP≧400 ng/ml (3C), tumor >3 cm (3D) and poor tumor grade (3E) in HCC.** Results are presented as individual and pooled odds ratio (OR), and 95% confidence interval (CI).

Eight studies reported the relationship between NLR and vascular invasion in HCC. High NLR tended to be correlated with the presence of vascular invasion in all studies, and a statistical significance was observed in three studies. Pooled data from all these eight studies showed a significant correlation between high NLR and the presence of vascular invasion (OR: 2.69, 95% CI: 2.01–3.59, P = 0.000; Figure 3A).Six studies reported the correlation between NLR and tumor multifocality in HCC. High NLR tended to be correlated with multiple tumors in all studies, and a statistical significance was observed in one study. Pooled data from all six studies showed a significant correlation between high NLR and the presence of multiple tumors (OR: 1.74, 95% CI: 1.30–2.34, P = 0.000; Figure 3B).Five studies reported the correlation between NLR and serum AFP level. High NLR tended to be correlated with higher incidence of AFP ≥ 400 ng/ml in four studies, and a statistical significance was observed in one study. Pooled data from all five studies showed a significant correlation between high NLR and higher incidence of AFP ≥ 400 ng/ml (OR: 1.46, 95% CI: 1.01–2.09, P = 0.04; Figure 3C).Five studies reported the relationship between NLR and tumor size in HCC. High NLR tended to be correlated with the presence of large tumor (>3 cm) in three studies, and a statistical significance was observed in one study. Pooled data from all five studies showed that high NLR tended to be correlated with higher incidence of tumor size >3 cm (OR: 1.35, 95% CI: 0.92–1.98, P = 0.13; Figure 3D).Three studies reported the relationship between NLR and tumor grade in HCC. High NLR tended to be correlated with poor tumor grade in two studies. Pooled data from all three studies showed that high NLR tended to be correlated with poor tumor grade (OR: 1.32, 95% CI: 0.69–2.52, P = 0.40; Figure 3E).

### Correlation between NLR cutoff value and OS in HCC

We compared the prognostic function of NLRs by using NLR cut-off values of 1.9, 3>NLR ≥ 2, 4>NLR ≥ 3, 4 and 5 reported in the included studies. The results indicated that all NLRs, except 3>NLR ≥ 2, were statistically correlated with poor OS of HCC (Table 2). Of these, an NLR of 5 was the most common use, with a pooled HR estimate of 2.87 (95% CI: 2.13–3.84, P = 0.000) from seven studies.

**Table 2:** Correlation between NLR cutoff value and OS in HCC

**Cut-off value of NLR**	**Number of study**	**HR**	**95% CI**	**p**	**Heterogeneity**		
					**X**^**2**^	**I**^**2**^	P*
**NLR = 5**	7	2.87	2.14–3.84	0.000	6.04	1%	0.42
**NLR = 4**	1	5.10	1.81–14.12	0.002	NA	NA	NA
**4>NLR ≥ 3**	2	2.32	1.58–3.42	0.000	0.29	0%	0.59
**3>NLR ≥ 2**	3	1.96	0.82–4.67	0.13	347.73	99%	0.000
**NLR = 1.9**	1	1.58	1.03–2.44	0.04	NA	NA	NA

NLR: neutrophil-lymphocyte ratio; HR: hazard ratio; CI: confidence interval; NA: not available. **“4>NLR ≥ 3”** means a NLR cutoff value greater than or equal to 3 but less than 4; **“3>NLR ≥ 2”** means a NLR cutoff value greater than or equal to 2 but less than 3.

### Publication bias

Publication bias estimate was mainly used to evaluate the reliability of meta-analysis results, especially which showed statistical significance [40]. Assessment of publication bias by using Egger’s test [41] (statistical significance was set at p <0.05) indicated that there were not significant publication bias in these studies.

## Discussion

NLR was often used to be a traditional inflammation marker, while its prognostic role in HCC was revealed just during the recent years. In this meta-analysis study, we mainly investigated the association between a high NLR and OS as well as DFS in HCC patients. The pooled outcomes in these cohorts demonstrated that high NLR significantly predicted poor OS of HCC patients initially treated either by curative therapies including liver transplantation, surgical resection and RFA or palliative treatments including TACE and mixed treatment. Furthermore, high NLR also significantly correlated with shortened DFS of patient treated by liver transplantation or surgical resection.

We also conducted pooled analysis on the correlation between high NLR and the pathologic features of HCC. The results indicated that high NLR was significantly correlated with the presence of vascular invasion, multiple tumors (satellite nodule) and high level of serum AFP (400 ≥ ng/ml). All of these 3 factors, especially vascular invasion and multiple tumors, have been documented to be the most powerful variables associated with HCC recurrence and compromise long-term survival [42]. Herein, high NLR is closely associated with more aggressive phenotype of HCC which is contributed to lower OS and DFS.

Although the results of the analysis are positive, the exact explanation for the observation that an elevated NLR among patients indicates an aggressive phenotype of tumor is not clearly defined. NLR is an inflammation marker. Most of the HCCs are related to chronic HBV or HCV infection, and the host would produce persistent chronic inflammation (hepatitis).The systemic and local inflammatory response to virus or tumor may provide a favorable microenvironment for tumor invasion and metastasis [43]. In addition, a high NLR was associated with a high infiltration of tumor-associated macrophages (TAMs), TAMs promote systemic neutrophilia via secreting cytokines such as IL-6 and IL-8 [24]. Furthermore, high expressions of granulocyte colony-stimulating factor in tumor tissue and macrophage colony-stimulating factor in peritumoral tissue are also associated with elevated circulating neutrophils [44-46]. Neutrophils are recognized as being the primary source of circulating vascular endothelial growth factor (VEGF), which has been established as a major contributor to tumor related angiogenesis, and hence increased the propensity of cancers metastasis [47]. On the other hand, tumor cells can reduce cytotoxic T lymphocyte (CTL) infiltration in the tumor by producing immunosuppressive cytokines such as vascular endothelial growth factor (VEGF), transforming growth factor–β (TGF-β), IL-10 and by consuming IL-2, a cytokine that is critical for maintaining CTL function [48]. Thus, NLR reflects an immune microenvironment that favors tumor vascular invasion and suppresses the host immune surveillance.

Our results should be interpreted cautiously since some limitations exist in this present meta-analysis. First, the cutoff value for defining high NLR has not been unified (2, 3, 4 or 5) in these studies, which leaded to between-study heterogeneity. Furthermore, different NLRs used in different centers make variety of data comparison and inconvenient for clinical use. Therefore, future large sample study to give a definitive cutoff value of NLR with good sensitivity and specificity is needed. Second, there was considerable clinical heterogeneity in the comparison of OS and DFS regarding different initial treatment, and thus, a meta-analysis of all included studies would not be appropriate. Third, these studies mainly focused on preoperative NLR, and the clinical significance of postoperative NLR change, which may dynamic reflect the change of balance between host inflammatory response and immune response after treatment, is largely unclear. Fourth, since our meta-analysis was carried out on the pooled data, strong recommendations at an individual patient level could not be obtained.

## Conclusions

In summary, the present meta-analysis provides coherent evidence that the elevated NLR is of strong prognostic significance in patients with HCC treated either by curative or palliative methods. Compared to other prognostic markers, NLR seems to be a convenient, easily-obtained and repeated, low cost and reliable predictor for HCC patients. A definitive cutoff value of NLR based on future large sample study is recommended. HCC patient with high NLR may benefit from anti-inflammatory treatment. Future research to test this hypothesis is necessary.

## Abbreviations

NLR: Neutrophil -lymphocyte ratio; HCC: Hepatocellular carcinoma; HR: Hazard ratio; CIs: Confidence intervals; OS: Overall survival; DFS: Disease free survival; LT: Liver transplantation; RFA: Radiofrequency ablation; TACE: Transarterial chemoembolization; CRP: C-reactive protein; SEs: Standard errors; TAMs: Tumor-associated macrophages; VEGF: Vascular endothelial growth factor; CTL: Cytotoxic T lymphocyte.

## Competing interests

The authors declare that they have no competing interests.

## Authors’ contributions

LSQ conceived and designed the review, supervised the data collection, statistical analysis and critically revised the manuscript. XWK and CD carried out the literature search, performed data extraction and data analysis, and wrote the manuscript. FSJ, PBG and LLJ participated in data extraction, and resolved the disagreement. All authors read and approved the final manuscript.

## Pre-publication history

The pre-publication history for this paper can be accessed here:

http://www.biomedcentral.com/1471-2407/14/117/prepub
